# The role of obinutuzumab in rituximab-refractory membranous nephropathy and minimal change disease

**DOI:** 10.1093/ckj/sfaf039

**Published:** 2025-02-08

**Authors:** Zewei Chen, Dechao Xu, Shuangcheng Wu, Wenyu Liu, Jianxiang Wu, Shengqiang Yu, Bing Dai, Zhiguo Mao, Xiang Gao

**Affiliations:** Department of Nephrology, Changzheng Hospital, Naval Medical University, Shanghai, China; Department of Nephrology, The First Navy Hospital of Southern Theater Command, Zhanjiang, Guangdong, China; Department of Nephrology, Changzheng Hospital, Naval Medical University, Shanghai, China; Department of Nephrology, Changzheng Hospital, Naval Medical University, Shanghai, China; Department of Nephrology, Changzheng Hospital, Naval Medical University, Shanghai, China; Department of Cardiology, Changzheng Hospital, Naval Medical University, Shanghai, China; Department of Nephrology, Changzheng Hospital, Naval Medical University, Shanghai, China; Department of Nephrology, Changzheng Hospital, Naval Medical University, Shanghai, China; Department of Nephrology, Changzheng Hospital, Naval Medical University, Shanghai, China; Department of Nephrology, Changzheng Hospital, Naval Medical University, Shanghai, China

**Keywords:** membranous nephropathy, minimal change disease, nephrotic syndrome, obinutuzumab, rituximab

## Abstract

**Background:**

Obinutuzumab, a new-generation anti-CD20 monoclonal antibody, was originally developed to overcome resistance to rituximab in B-cell malignancies. There is limited research regarding the use of obinutuzumab in patients with rituximab-refractory membranous nephropathy (MN) and minimal change disease (MCD).

**Methods:**

A retrospective analysis was performed at Changzheng Hospital from September 2022 to September 2024, and screened patients with rituximab-refractory MN or MCD. Participants were treated because they were refractory to rituximab and consented to receive infusions of obinutuzumab. Primary outcomes were defined as complete remission (CR, proteinuria <0.3 g/d) or partial remission (PR, proteinuria <3.5 g/d with a ≥50% reduction). Secondary outcome was immunological remission in patients with phospholipase A2 receptor (PLA2R)-related MN.

**Results:**

Seven patients with MN and five with MCD were included in the cohort. Among patients with MN, six of seven (86%) achieved at least PR, of whom two patients reached CR with a median time to first remission (either PR or CR) of 8.0 months. Among patients with positive serum anti-PLA2R antibodies at baseline, all achieved an immunological response. No patients experienced a relapse during the follow-up period. Among patients with MCD, all patients achieved a CR with the median time of 1.0 months. Patients who were steroid-dependent or immunosuppressant-dependent were able to taper their medications in the short term without experiencing relapse. No treatment-related severe adverse events were reported.

**Conclusions:**

Our study demonstrated that obinutuzumab represents a promising alternative therapeutic option for the management of rituximab-refractory MN and MCD.

## Graphical Abstract

**Graphical Abstract:**
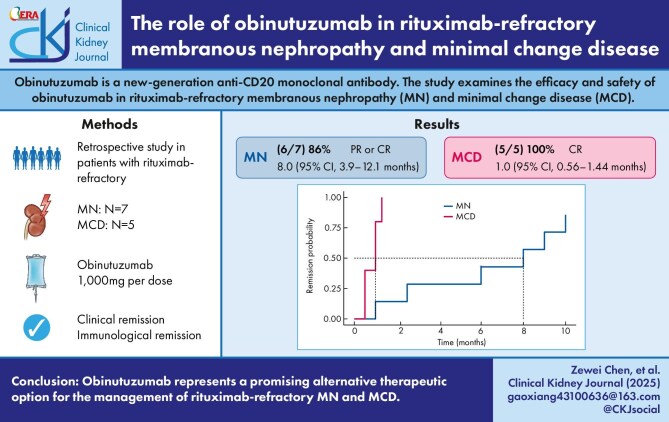

KEY LEARNING POINTS **What was known:**

- Rituximab, a chimeric, monoclonal anti-CD20 antibody, is increasingly used in the management of membranous nephropathy (MN) and minimal change disease (MCD).
- Obinutuzumab is a new-generation anti-CD20 antibody that was initially developed to address rituximab resistance in B-cell malignancies.
- Obinutuzumab's effect in patients with rituximab-refractory MN and MCD is unclear.

**This study adds:**

- Anti-rituximab antibodies were positive in some participants with rituximab-refractory MN and MCD.
- Treatment with obinutuzumab will induce clinical remission and immunological remission in patients with rituximab-refractory MN and MCD.

**Potential impact:**

- Given its effectiveness and acceptable safety profile, obinutuzumab should serve as a promising alternative therapeutic option in patients with rituximab-refractory MN and MCD.

## INTRODUCTION

Membranous nephropathy (MN) and minimal change disease (MCD) are the leading causes of primary nephrotic syndrome (NS) in adults and children [1]. The primary objective of treatment is to maintain renal function and avert the necessity for kidney replacement therapy while concurrently minimizing the toxicities of therapy [2]. Nonetheless, in individuals suffering from persistent NS who either do not achieve remission or experience frequent relapses, there is an escalating risk of progressive renal function decline [3].

B lymphocytes have been shown to play a crucial role in the pathogenesis underlying MN and MCD [4, 5]. Anti-CD20 monoclonal antibodies, such as rituximab, can enable effective depletion of B-cells through selectively targeting them, thereby selectively targeting antibody production [4]. Rituximab was developed as a first-line regimen for patients with MN and MCD who are at risk of progressing to renal failure or exhibiting persistent nephrotic syndrome despite receiving conservative management [6, 7]. Meanwhile, rituximab has been reported to safely reduce the annual relapse rate in most studies, with some cases achieving long-term remission However, despite its demonstrated efficacy, previous studies indicated that 20%–40% patients with MN [8, 9] and 5%–10% patients with MCD [10] failed to respond to rituximab treatment and continued to remain nephrotic. As a chimeric monoclonal antibody, rituximab poses a risk of eliciting the formation of anti-drug antibodies. The detection of anti-rituximab antibodies has been documented in patients with MN and MCD undergoing repeated rituximab infusions, which may diminish circulating levels of rituximab and lead to shorter or absence of B-cell depletion [11, 12].

Obinutuzumab is a humanized and glycoengineered, type II anti-CD20 monoclonal antibody that was originally developed to address resistance to rituximab in B-cell malignancies [13]. Increasing evidence suggests that obinutuzumab may serve as a more potent and enduring B-cell depleting agent compared with traditional treatments [14–19]. The potential application of obinutuzumab as an alternative therapeutic option for patients with MN and MCD has emerged as a novel area of exploration within the field. However, to date there has been limited research regarding the efficacy and safety of obinutuzumab in treating MN and MCD, especially in rituximab-refractory patients. We conducted a retrospective cohort study and aimed to assess the efficacy and safety of obinutuzumab in patients with MN or MCD who failed to respond to rituximab-based regimens.

## MATERIALS AND METHODS

### Study design and patients

In our retrospective cohort we screened all consecutive participants diagnosed with rituximab-refractory MN or MCD who received at least one dose of obinutuzumab therapy during hospitalization at the Kidney Institute of Changzheng Hospital from 1 September 2022. Rituximab-refractory MN was defined by the persistent nephrotic syndrome, indicated by low levels of serum albumin or sustained detection of unchanged or high levels of anti-phospholipase A2 receptor (PLA2R) antibodies for a period exceeding 6 months, as determined by physician discretion, following the administration of rituximab. Patients with MCD who experienced consecutive relapses during the tapering or withdrawal of steroids or immunosuppressants following rituximab treatment were also categorized as rituximab-refractory MCD. The administration of rituximab was based on physician preference according to two separate dosing regimens: either 375 mg/m^2^ of body surface intravenously weekly for 4 weeks, or 1 g on days 1 and 15. A second course of rituximab was given 6 months later, contingent upon B-cell count and proteinuria levels.

The exclusion criteria included nephropathy secondary to autoimmune diseases, malignancies, infectious diseases, or pharmacological agents. Individuals with a previous history of kidney transplantation or dialysis were excluded. In addition, participants with acute or chronic infections necessitating therapies or with seropositivity for hepatitis B surface antigen or hepatitis C were also excluded from the cohort. The study was in compliance with the Helsinki Declaration and received approval from the Ethics Committee of Changzheng Hospital. Informed consent was obtained from all patients.

### Therapy

The infusion interval between rituximab and obinutuzumab was maintained at a minimum of 6 months. All patients were hospitalized for administration of obinutuzumab. Each obinutuzumab infusion was administered at a dosage of 1000 mg [20]. The frequency of obinutuzumab infusions was determined collaboratively between the physicians and the patients according to professional consultation. Obinutuzumab was first diluted using 500 mL of normal saline. Participants were administered an intravenous injection of 5 mg dexamethasone or 40 mg methylprednisolone, along with an intramuscular injection of 12.5 mg promethazine hydrochloride as systematic premedication. The infusion was monitored at a rate of 25 mL/h within the initial 30 minutes, the rate being gradually increased every 30 minutes to the goal of 200 mL/h, depending on patient tolerance.

### Data collection

Patient records were retrospectively reviewed to collect demographic and clinical data. The baseline was defined as first hospital admission for obinutuzumab therapy. Demographic data included gender, and age at baseline. Serum creatinine (SCr), serum albumin, estimated glomerular filtration rate (eGFR), 24-hour urine protein excretion, CD cells, anti-PLA2R antibodies, and anti-rituximab antibodies were recorded as laboratory parameters. The eGFR was calculated by using the Chronic Kidney Disease Epidemiology Collaboration (CKD-EPI) formula [21]. B-cell depletion was defined as absolute CD19-positive count <5 cells/µL, while B-cell reconstitution was defined as absolute CD19-positive count >5 cells/µL through peripheral blood flow cytometry. The titer of serum anti-PLA2R antibodies was analyzed with a minimum detectable threshold of 14 RU/mL. Enzyme-linked immunosorbent assay (ELISA) findings were interpreted as follows: <14 RU/mL, negative; 14–20 RU/mL, borderline; and >20 RU/mL, positive. The titer of anti-rituximab antibodies was detected by ELISA according to the manufacturer's instructions (< 1 ng/mL, negative; >1 ng/mL, positive; Shikari^®^ S-ATR, IBL International GmbH). All data were systematically obtained via the hospital's electronic medical record systems and continuous interviews performed by telephone. Each patient was evaluated individually by our nephrologists based on history, physical and laboratory testing, and kidney biopsy.

### Outcomes and definitions

The follow-up observations were made from the initiation of obinutuzumab treatment until the date of the participants’ final visit within the study period. All participants were monitored for at least 6 months post-baseline. The final follow-up time was September 2024.

Patients were designated to achieve a complete remission (CR) or partial remission (PR) if there was an observed improvement in kidney parameters at any point subsequent to the initiation of therapy based on the observation that patients may continue to experience improvement in kidney parameters even after treatment is completed. CR was defined as a 24-hour urinary protein excretion of <0.3 g/d, with a normal serum albumin level and a stable SCr level. PR was defined as a 24-hour urinary protein excretion of >0.3 g/d and <3.5 g/d, with a ≥50% reduction from peak values accompanied by an improvement or normalization of the serum albumin level and a stable SCr. Time from treatment to a PR or CR was also noted. Patients were deemed to have no response (NR) if they did not fulfill the criteria for either CR or PR. Relapse was defined as a deterioration in proteinuria following the attainment of PR or CR. Immunological outcomes were evaluated exclusively in cases with PLA2R-related MN. Immunological remission was defined as the reduction of serum anti-PLA2R antibodies levels to 14 RU/mL in PLA2R-associated MN [22].

### Safety evaluation

Safety outcomes included adverse and serious adverse events recorded during the infusions of obinutuzumab and throughout the follow-up period. Serious adverse events covered organ or life-threatening events, or requirement for additional hospitalization.

### Statistical analysis

Quantitative data that conformed to the normal distribution were expressed as mean ± standard deviation, while non-normally distributed data were presented as median (interquartile range [IQR]). A *t*-test was performed to assess statistical significance. Categorical data were represented as number (percentage). Kaplan–Meier analysis was used for plotting and estimation of remission probabilities after obinutuzumab in all patients. The R packages ‘survival’, ‘survminer’, and ‘ggplot2’ were run in RStudio 4.4.1. For statistical analysis SPSS version 26.0 was used.

## RESULTS

### Patient population and baseline characteristics

We identified a cohort of 12 patients, including 7 with MN and 5 with MCD, who were treated with obinutuzumab after not responding to a rituximab-based regimen, as detailed in Table 1. Prior to initiation of obinutuzumab, the majority of these patients underwent multiple immunomodulatory therapies in addition to rituximab, all of which proved unsuccessful.

**Table 1:** Baseline characteristics of patients at the time of initial obinutuzumab treatment.

Patient	Sex/age (years)	Duration (months)^a^	Previous therapy	Dosage of RTX	Proteinuria (g/24 h)	Albumin (g/L)	SCr (µmol/L)	eGFR (mL/min)	CD19 B cells (%, cell/µL)	Anti-PLA2R Ab (RU/mL)	Anti-RTX Ab (ng/mL)
Group 1: MN											
1	M/52	36	PRED/TAC (6 m)/CY (4.8 g)/RTX (3 g)	1 g (0, 0.5, 6 m)	8.15	25.4	121	62.1	6.1% (78)	21.77	Not detected
2	F/68	8	RTX (2 g)	1 g (0, 0.5 m)	6.02	17.3	68	84.1	1.2% (18)	234.29	Not detected
3	M/63	25	PRED/TAC (6 m)/CY (6.8 g)/RTX (2.4 g)	0.6 g (0, 1 w, 2 w, 3 w)	8.99	16.9	143	44.5	0.5% (3)	141.35	0
4	M/58	23	CSA (6 m)/CY (3.4 g)/RTX (3 g)	1 g (0, 0.5 m, 6 m)	14.6	22.6	135	49.5	0	86.15	0
5	M/56	13	PRED/CSA (6 m)/RTX (2 g)	1 g (0, 0.5 m)	20.4	20.4	98	73.9	1.4% (19)	100.91	73.19344
6	M/42	15	TAC (6 m)/RTX (2 g)	1 g (0, 0.5 m)	27.8	24.3	199.4	34.6	0.3% (4)	2.65	Not detected
7	F/71	93	PRED/CSA (6 m)/CY (11.4 g)/RTX (2 g)	1 g (0, 0.5 m)	5.4	28.9	110	43.6	0.5% (9)	<2	2.391496
Group 2: MCD											
8	M/16	7	PRED-dependent (15 mg)/RTX (2 g)	1 g (0, 0.5 m)	3.39	31.7	70	147.1	0% (0)	None	0.1678
9	M/20	24	PRED (15 mg)/TAC-dependent (1 mg/bid)/RTX (3 g)	1 g (0, 0.5 m, 6 m)	1.49	35.8	63	162.5	0.3% (3)	None	0.684735
10	M/52	46	PRED (20 mg)/TAC-dependent (0.5 mg/qd)/CY (8 g)/RTX (2.4 g)	0.6 g (0, 1 w, 2 w, 3 w)	3.23	38.2	78	100.2	8.9% (154)	None	Not detected
11	F/26	13	PRED (15 mg)/TAC-dependent (0.5 mg/bid)/RTX (2 g)	1 g (0, 0.5 m)	3.84	33	54	125.5	7.5% (276)	None	0.337375
12	M/17	8	PRED-dependent (20 mg)/RTX (2 g)	1 g (0, 0.5 m)	0.88	37	54	146.7	0.5% (8)	None	28.672

eGFR is expressed as mL/min per 1.73 m^2^ calculated by the CKD-EPI equation.

Ab, antibody; RTX, rituximab antibody; M, male; F, female; PRED, prednisone; CY, cyclophosphamide; CSA, cyclosporin; TAC, tacrolimus; RTX, rituximab; m, month; w, week.

a Time from kidney biopsy to obinutuzumab administration.

Among the seven patients with MN, the mean age was 58.6 years (range 42–71), and 71% of these patients were male. The median time from first kidney biopsy to obinutuzumab administration was 23 months, with an IQR of 13–36 months. Among the patients studied, six (86%) had a previous history of calcineurin inhibitor use and four (57%) had previously used cyclophosphamide. In relation to regimens based on rituximab, all patients were transitioned to obinutuzumab due to unsatisfactory response following rituximab therapy with an average dose of 2.3 g. At the time of enrollment, the median 24-hour urinary protein excretion was 8.9 g/24 h (IQR 6.0–20.4), the mean serum albumin was 22.3 ± 4.4 g/L, and the median eGFR was 49.5 mL/min (IQR 43.6–73.9). Three patients (43%) retained B-cell depletion during the follow-up after rituximab infusion, while four patients (57%) exhibited evidence of CD19 cell reconstitution. Concerning anti-PLA2R antibodies, five patients (71%) demonstrated titers of serum anti-PLA2R antibodies of at least 20 RU/mL. Of the seven patients, four (57%) were tested for anti-rituximab antibodies, of which two (patients 5 and 7) were positive (>1 ng/mL) while two were negative (<1 ng/mL).

Among the five patients with MCD, the mean age was 26 years (range 17–39), with 80% of the patients being male. The median time from kidney biopsy to obinutuzumab administration was 13 months (IQR 7.5–35 months). Regarding previous application of rituximab, two patients (patients 8 and 12) transferred to obinutuzumab due to steroid-dependence after rituximab treatment, while three others (patients 9, 10, and 11) switched to obinutuzumab because of immunosuppressant-dependence (tacrolimus). The average dose of rituximab was 2.3 g. At baseline, the median 24-hour urinary protein excretion was 3.2 g/24 h (IQR 1.2–3.6), the mean serum albumin level was 35.1 ± 2.7 g/L, and the mean eGFR was 136.4 ± 24.1 mL/min. Three patients (patients 10, 11, and 12) had evidence of B-cell reconstitution during the follow-up period after administration of rituximab. Anti-rituximab antibody detection was conducted in four patients (80%), of which one (patient 12) was positive and three were negative.

### Outcome

Overall, all patients received at least one infusion of obinutuzumab, with 11 (92%) achieving either PR or CR (Table 2). Detailed information is shown in Supplementary Fig. S1.

**Table 2:** Outcomes of patients who received obinutuzumab therapy.

			Last follow-up (months)						Response		Time to response (months)		
Patients	Obinutuzumab therapy	Other immunosuppressive therapy	Time (months)	Proteinuria (g/24 h)	Albumin (g/L)	SCr (µmol/L)	eGFR (mL/min)	Adverse events	Immunological response	Clinical response	PR	CR	Relapse
Group 1: MN													
1	1 g (0, 6 m, 12 m, 18 m)	None	20	1.98	39	105	70.0	None	Remission (<2 RU/mL)	PR	8		
2	1 g (0, 0.5 m)	None	8	2.14	33.8	59.2	89.9	Rash/itching	Remission (<2 RU/mL)	PR	2.5		
3	1 g (0, 0.5 m, 6 m, 6.5 m)	None	18	0.21	43.2	148	42.7	None	Remission (<2 RU/mL)	CR	9	18	
4	1 g (0, 6 m, 12 m,18 m)	None	24	0.73	44.8	117	58.9	None	Remission (<2 RU/mL)	PR	10		
5	1 g (0, 0.5 m,6 m)	None	6	8.25	21.7	66	119.2	None	Remission (5.18 RU/mL)	NR			
6	1 g (0, 0.5 m)	None	6	3.43	35.8	142	52.1	None	Remission (<2 RU/mL)	PR	6		
7	1 g (0)	None	7	0.16	45.5	106	45.6	None	Remission (<2 RU/mL)	CR	1.0	7	
Group 2: MCD													
8	1 g (0, 0.5 m)	PRED (20 mg, 2 m)	12	0.03	45	88.4	110.9	None		CR	NA	0.5	
9	1 g (0)	TAC (1 mg/bid, 2 m)	7	0.03	44	63	162.5	None		CR	NA	1.0	
10	1 g (0, 0.5 m)	TAC (0.5 mg/qd, 1 m)	12	3.62	21.8	68	118.3	None		CR	NA	1.3	12
11	1 g (0)	TAC (0.5 mg/bid, 1 m)	6	0.06	42.5	49	129.5	None		CR	NA	1.0	
12	1 g (0, 0.5 m, 6 m)	PRED (20 mg, 1.5 m)	12	0.1	38	66	135.2	None		CR	NA	0.5	

PRED, prednisone; TAC, tacrolimus; m, month; NA, data not available.

**Figure 1:**
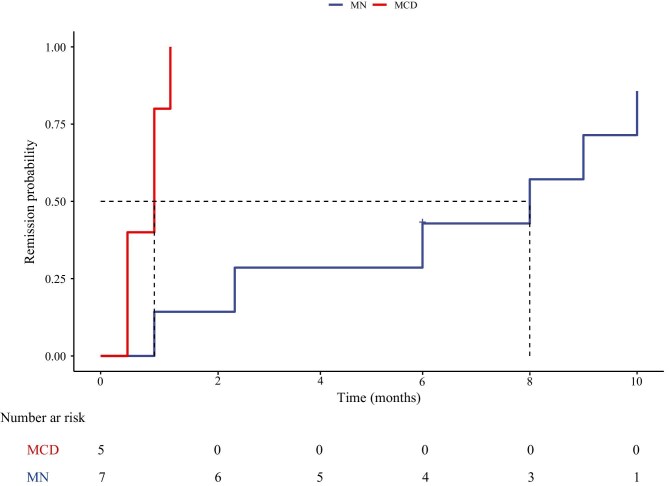
Probability of achieving remission (either partial or complete) after obinutuzumab infusion.

Among the seven patients with MN, the average dose of obinutuzumab was 2.9 g. The median follow-up time was 8 months (IQR 6–20). At the final follow-up time, the median proteinuria decreased from 8.9 g/24 h (IQR 6.0–20.4) to 1.98 g/24 h (IQR 0.21–3.43) (*P* = .006), whereas median serum albumin increased from 22.3 ± 4.4 g/L to 37.7 ± 8.3 g/L (*P* < .001). Renal function showed improvement in six patients (86%), as evidenced by an increase in the median eGFR, which increased from 49.5 mL/min (IQR 43.6–73.9) to 58.9 mL/min (IQR 45.6–89.9) (*P* < .001). Among the five patients with positive titers of serum anti-PLA2R antibodies at baseline, all achieved an immunological response represented by abolishment of anti-PLA2R antibody titer. Six patients (86%) achieved at least PR during the follow-up period, of whom two (patients 3 and 7) had reached CR. Only one patient (patient 5) failed to achieve remission during the follow-up; yet his proteinuria dropped from 20.44 to 8.25 g/24 h at 6 months, with the anti-PLA2R antibodies titer dropping from 100.91 to 5.18 RU/mL. The median time to first remission from the initial infusion of obinutuzumab (either PR or CR) was 8.0 months. Kaplan–Meier analysis of the cohort is presented in Fig. 1. The remission rates at 6 and 12 months were 3 (43%) and 6 (86%), respectively. No relapse was detected in the follow-up period. Only one patient (patient 2) experienced a mild infusion-related reaction, rash and itching, and no severe complication was observed.

In the five patients with MCD, each patient received one to three infusions of obinutuzumab, with an average of 1.8 g. Among the patients who were steroid-dependent (patients 8 and 12), corticosteroid was gradually tapered and maintained at a minimal dose after receiving obinutuzumab therapy, with no relapse observed during the follow-up period; for patients who were immunosuppressant-dependent (patients 9, 10, and 11), tacrolimus was tapered and discontinued within 1 month without relapse (detailed in Supplementary Fig. S1). The median follow-up time was 12 months (IQR 6.5–12). At the last follow-up, the median proteinuria dropped significantly from 3.2 g/24 h (IQR 1.2–3.6) to 0.06 g/24 h (IQR 0.03–1.86) (*P* = .012), whereas median serum albumin increased from 35.1 ± 2.7 g/L to 38.3 ± 9.6 g/L (*P* < .001). All five patients achieved a CR, with a median time to CR after the initial infusions of obinutuzumab being 1.0 months (95% CI 0.56–1.44) (Fig. 1). One patient (patient 10) experienced a relapse with proteinuria rising to 3.62 g/24 h after maintaining remission for 12 months. No infusion-related reactions were reported in five patients.

## DISCUSSION

This retrospective analysis demonstrated that the infusions of obinutuzumab can induce both immunological and clinical remission in patients with rituximab-refractory MN or MCD. Using this approach, six of seven (86%) treated patients with MN experienced PR, two of them experiencing CR and one non-responder exhibiting a decreasing trend in proteinuria. Despite the presence of progressive chronicity or kidney dysfunction in patients with MN, the response to obinutuzumab therapy was favorable, resulting in improved renal function. Meanwhile, patients with MCD achieved an overall CR within 1.3 months. Furthermore, the safety of obinutuzumab was acceptable in this study.

Rituximab, as a chimeric monoclonal antibody, is composed of a murine-derived anti-human CD20 variable region and human IgG1 constant regions [23]. The application of chimeric monoclonal antibodies may be complicated by the emergence of anti-drug antibodies such as anti-rituximab antibodies [24]. The occurrence of anti-rituximab antibodies is not uncommon, with 23% of patients with MN and 29% with MCD patients treated with rituximab developing these antibodies during the follow-up [25, 26]. These antibodies neutralize the therapeutic activities of rituximab (complement-dependent cytotoxicity and antibody-dependent cell-mediated cytotoxicity) in 80% of patients, leading to an accelerated B-cell reconstitution and an increased rate of relapse [26]. Obinutuzumab, as a humanized antibody, may possess the potential to enhance efficacy by minimizing the risk of under-activity resulting from the production of anti-drug antibodies [27]. It is therefore pertinent to assess antibody formation against rituximab in cases of inadequate treatment response and to be cautious with successive infusions if anti-rituximab antibodies are detected. In such a case, treatment with another anti-CD20 antibody such as obinutuzumab could be considered. However, due to the degree of homology between rituximab and obinutuzumab, there is potential for cross-reactivity, which may provoke similar or equally strong effects following successive infusions of obinutuzumab [28, 29]. Luckily, previous research demonstrated that anti-rituximab antibodies exhibited cross-reactivity with obinutuzumab in only 20% of patients, indicating that obinutuzumab may be safely applied as an alternative to rituximab [26]. In our cohort, among the three patients who tested positive for anti-rituximab antibodies, two (67%) patients achieved clinical remission, despite the other patient with a high titer of anti-rituximab antibodies failing to attain clinical remission during the follow-up period; yet his proteinuria and the anti-PLA2R antibodies was on an improving trend during limited follow-up. Currently, the assessment of anti-rituximab antibodies is not routinely performed in patients with rituximab resistance, and there is a need for future large-sample clinical cohort studies to evaluate the guiding value of anti-rituximab antibodies for novel CD20 antibodies such as obinutuzumab.

There are currently no guidelines for a clinical standard dosage and frequency of obinutuzumab for the treatment of MN and MCD due to the finiteness of sample data. Nevertheless, recent trials in refractory MN have reached a consensus that obinutuzumab infusions are based on a dosage of 1000 mg per administration, and the time interval between the first and second infusions typically spans 2–3 weeks [14–16, 22]. Under this dosage, both the remission rates and the safety profile have been deemed satisfactory. Regarding the additional doses of obinutuzumab infusions, a personalized treatment prescription was selected, which primarily involves the following considerations. Firstly, B lymphocytes were considered to be an important indicator in the prediction of treatment response to obinutuzumab in patients. Recent studies have corroborated that low-dose infusions of obinutuzumab can induce more prolonged and extensive B-cell depletion than rituximab in patients with refractory nephrotic syndrome [16, 17]. Su *et al*. [22] also demonstrated that the median absolute B-cell count in patients treated with obinutuzumab remained <5 cells/µL even after 7 months, indicating a sustained B-cell depletion. This provided evidence that the interval between the second and third infusions exceeded 6 months, based on B-cell counts. Specifically, three patients (patients 1, 4, and 5) did not achieve complete B-cell depletion at the 6-month follow-up (116, 19, and 47 cells/µL, respectively; Supplementary Table S1). Consequently, an additional infusion of obinutuzumab was administered. Secondly, the efficacy of the drug is an important point to consider. Two patients (patients 3 and 5) failed to achieve PR with a high level of proteinuria (>3.5 g/24 h) at the 6-month follow-up (Supplementary Fig. S1), prompting the administration of an additional dose. However, extensive immunological damage necessitates prolonged podocyte remodeling before the architecture and function of the glomerular filtration barrier are restored in MN. We cannot rule out that extending the follow-up duration in patients who did not have a remission at 6 months might eventually have increased the percentage of patients exhibiting a response [30]. Furthermore, anti-PLA2R antibodies levels predict response. High levels of anti-PLA2R antibodies were correlated with a lower likelihood of remission [31]. Qualitative analyses of anti-PLA2R antibodies during the course of disease already revealed that the disappearance of antibodies preceded the onset of clinical response. The persistence of antibodies at the end of therapy predicts an unfavorable outcome, indicating that the immunological remission could become the goal of therapy [32]. In our study we observed that the immunological response to obinutuzumab preceded the clinical response in all patients with positive titers of anti-PLA2R antibodies. In patient 5 who failed to achieve clinical remission, an elevated serum anti-PLA2R antibody level was noted following rituximab therapy, while the levels showed a significant reduction after infusions of obinutuzumab. Despite patient 5 reaching an immunological response, his anti-PLA2R antibody levels were not entirely eradicated (<2 RU/mL), so we performed an additional obinutuzumab infusion.

MCD, as another common form of NS, presents significant challenges due to its high relapse rates. Notably, individuals with MCD who experience frequently relapsing or steroid-dependent NS during childhood or adulthood are at an increased risk for long-term toxicity [33]. CD20 monoclonal antibody has proved to be an effective and safe therapeutic option for patients with MCD in sustaining the state of remission and minimizing corticosteroid or immunosuppressant exposure [34]. In our study, we speculate on potential reasons for the poor performance of rituximab in patients with MCD. On the one hand, three patients (patients 8, 11, and 12) had a history of previous COVID-19 infection. As reported in the literature, doses of COVID-19 vaccines have been shown to elicit a potent immune response, effectively activating T- and B-cell inflammatory cytokines, which may impact the efficacy of rituximab [35]. On the other hand, steroid or tacrolimus tapering in all patients with MCD led to recurrence or worsening after rituximab therapy; yet with the transition to obinutuzumab patients were able to taper their immunosuppressants without experiencing relapse in the short term. This also explains why patients with proteinuria levels of 0.88 g/24 h transferred to treatment with obinutuzumab. The observed results revealed that five patients with MCD achieved a CR within 1.3 months, but one patient experienced a relapse, with proteinuria increasing to 3.62 g/24 h after maintaining remission for 1 year. In previous Asian studies, relapses in MCD patients treated with rituximab often occurred within the first year, and rituximab was given at 6 months apart from a consolidation therapy [36]. Therefore, whether the optional courses of obinutuzumab consolidation at 6 or 12 month s to sustain long-term remission in patients with MCD needs to be investigated in future studies' to 'whether optional courses of obinutuzumab consolidation at 6 or 12 months are necessary to sustain long-term remission in patients with MCD needs to be investigated in future studies.

Notwithstanding these strengths, our series has significant limitations; as with most previous retrospective research, there were missing or incomplete data and risks of selectivity bias or recall bias. Meanwhile, the sample size of the current study is small. Finally, we did not compare the dynamic changes in B lymphocytes at different periods after the administration of obinutuzumab. Consequently, the investigation into the association between B-cell depletion or reconstitution and clinical outcome remained constrained.

In conclusion, we were able to demonstrate that obinutuzumab is both effective and safe in treating patients with MN and MCD refractory to rituximab, thereby providing a viable alternative therapeutic option for these individuals.

## Supplementary Material

sfaf039_Supplemental_Files

## Data Availability

The data underlying this article will be shared upon reasonable request to the corresponding author.
