# Supplementary material for: The role of obinutuzumab in rituximab-refractory membranous nephropathy and minimal change disease
Source: Clin Kidney J. 2025 Feb 8;18(3):sfaf039. doi: 10.1093/ckj/sfaf039 (PMC11914879; doi:10.1093/ckj/sfaf039)
Supplement: sfaf039_Supplemental_Files [file sfaf039_supplemental_files.zip › Supplementary Figures.pdf]

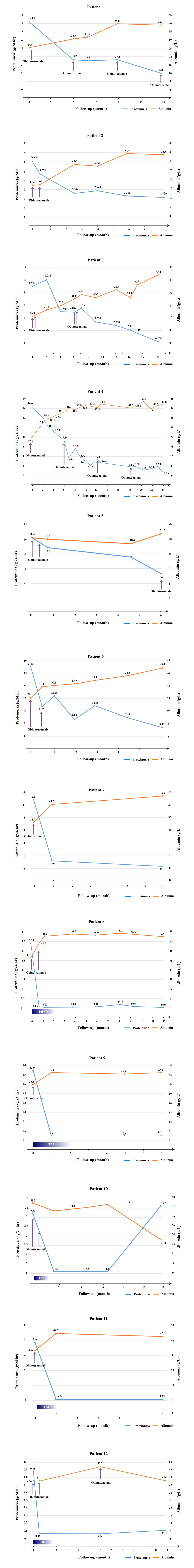

Supplementary Figure 1. Proteinuria and serum albumin trend after Obinutuzumab in all patients. PRED, prednisone; TAC, tacrolimus.
