# Supplementary material for: The role of obinutuzumab in rituximab-refractory membranous nephropathy and minimal change disease
Source: Clin Kidney J. 2025 Feb 8;18(3):sfaf039. doi: 10.1093/ckj/sfaf039 (PMC11914879; doi:10.1093/ckj/sfaf039)
Supplement: sfaf039_Supplemental_Files [file sfaf039_supplemental_files.zip › Supplementary Table.docx]

Table 3. B-cell levels at 6-month follow after Obinutuzumab treatment.

| Patients | CD19 B cell (%) | CD19 B cell (cell /µl) |  |
| --- | --- | --- | --- |
| Group 1: MN | |  |  |
| 1 | 8.9 | 116 |  |
| 2 | 0 | 0 |  |
| 3 | 0 | 0 |  |
| 4 | 1.3 | 29 |  |
| 5 | 3.3 | 47 |  |
| 6 | 0 | 0 |  |
| 7 | 0 | 0 |  |
| Group 2: MCD | | | |
| 8 | 0 | 0 |  |
| 9 | 0 | 0 |  |
| 10 | 0 | 0 |  |
| 11 | 0 | 0 |  |
| 12 | 0 | 0 |  |

MN, membranous nephropathy; MCD, minimal change disease.
